# Comparative Review of Outcomes of Totally Extraperitoneal (TEP) and Transabdominal Preperitoneal (TAPP) Primary Inguinal Hernia Repair

**DOI:** 10.7759/cureus.49790

**Published:** 2023-12-01

**Authors:** Martin Verheij, Alaa E Abdalla, Pradeep Chandran

**Affiliations:** 1 Trauma Surgery, Kings College Hospital, London, GBR; 2 General Surgery, Mediclinic Parkview Hospital, Dubai, ARE; 3 Trauma Surgery, King's College Hospital, London, GBR

**Keywords:** postoperative pain, tep, tapp, cosmetic outcome, totally extraperitoneal, transabdominal preperitoneal

## Abstract

Introduction: There is an ongoing debate about the efficacy and postoperative outcomes of transabdominal preperitoneal (TAPP) and totally extraperitoneal (TEP) inguinal hernia repair. Our aim is to assess the surgical outcomes of each technique, focusing predominantly on postoperative components to determine if establishing a policy to advocate for a single technique is warranted.

Method: A literary review of randomized control trials and cohort studies to delineate recurrent concerns or points of contention was undertaken. A retrospective, comparative analysis was performed of TEP and TAPP primary inguinal hernia repairs performed by surgeons with more than five-year experience with their preferred technique over a three-year period (January 2020 to December 2022) at three separate institutions.

Results: A total of 279 applicable cases were reviewed of which 38% (n=106) were performed as TEP and 62% (n=173) performed as TAPP. The demographic of the cohort was heavily skewed towards the male population as expected; however, there were no differences between each subgroup. TEP hernia repair showed a significantly improved postoperative pain score at one and 24 hours, respectively (1.67 ± 0.45, p < 0.05 and 1.97 ± 0.31, p < 0.05). No discernible difference was noted in the categories of length of hospital stay, recurrence rate, and overall patient satisfaction.

Conclusion: The study showed overall improved results using the TEP inguinal hernia repair technique; however, no statistically significant results were demonstrated in the long term to advocate for changes to pre-existing surgeon preferences.

## Introduction

Inguinal hernias are a very common surgical presentation affecting both genders with a prevalence of 7.7% for all ages and the highest prevalence of 12.7% occurring in the Asian subgroup [1]. Males have a higher lifetime risk of inguinal hernias estimated at 32.9% compared to women with a significantly reduced 12.9% risk [2]. Despite the ubiquity of inguinal hernias, there is a lack of standardization in the technique of hernia repair. There is a wide range of techniques for repair at a surgeon’s disposal ranging from open to laparoscopic and there is an ongoing debate about the best techniques for inguinal hernia repair. Current outcome goals sought for any inguinal hernia surgery are a reduction in reoccurrence rates, postoperative pain, and improvement of the patient’s quality of life.

Mesh repair techniques (open or laparoscopic) are a popular choice for surgeons as they are excellent for reinforcement of the abdominal wall and they are associated with the lowest reoccurrence rates [3]. From an open approach point of view, the Lichtenstein technique is regarded as the standard technique popularized by a higher efficacy and a low hernia reoccurrence rate. However, it’s associated with higher rates of chronic pain [4]. Alternatively, laparoscopic mesh repair techniques can be employed and these aim at minimizing postoperative pain and shortening recovery without any compromise in the reoccurrence rate. Transabdominal preperitoneal (TAPP) and totally extraperitoneal (TEP) are the principal laparoscopic techniques in use today around the world.

The predominant difference between these two techniques, as the names suggest, is access to the peritoneum during the process of inguinal hernia repair. The TAPP inguinal hernia repair consists of a conventional laparoscopic approach with repair taking place through the peritoneum from the abdominal cavity. The TEP inguinal hernia repair requires the placement of a laparoscopic camera in the preperitoneal space with balloon dilatation taking place posterior to the rectus muscle and anterior to the transversalis fascia.

Various studies prior have compared the efficacy of the three techniques: TAPP, TEPP, and open repair, through randomized controlled trials (RTC) and retrospective analysis of registries. This study will aim to investigate the benefit of each laparoscopic technique, TAPP, and TEP, in the categories of length of hospital stay, postoperative pain, patient satisfaction, recurrence rate, and cosmetic outcome. The intention is to determine if there is a discernible difference in benefit to the patient and, if any difference does exist, does it warrant a change in current practice to adopt the more advantageous technique.

## Materials and methods

**Figure 1:**
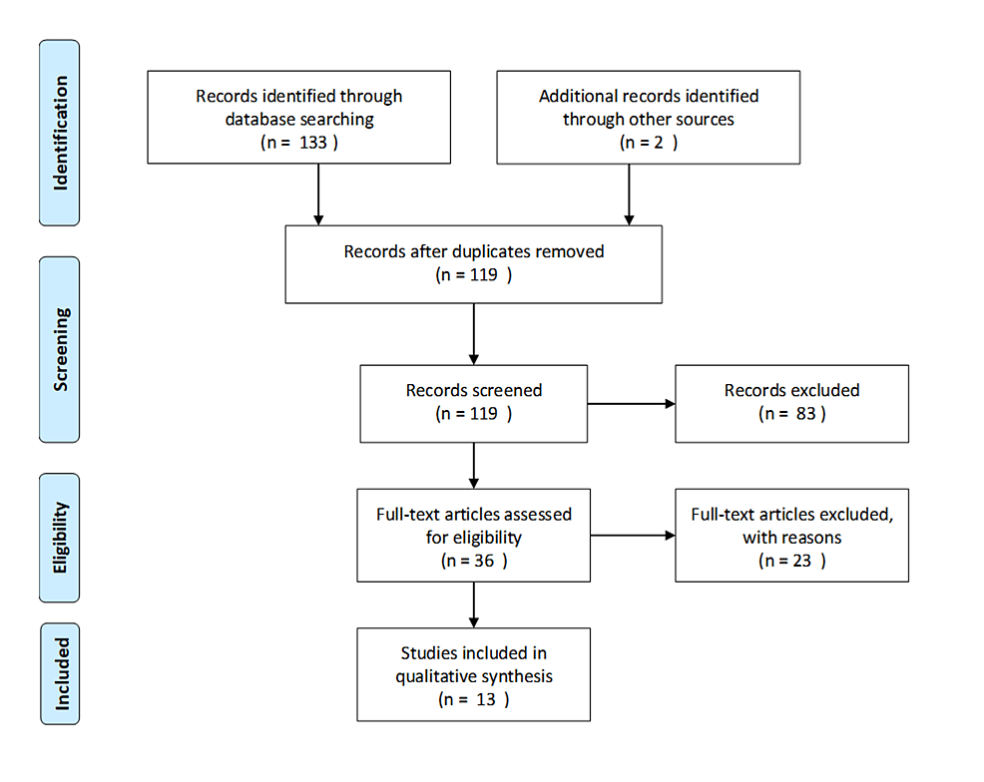
PRISMA algorithm outlining the inclusion and exclusion process of relevant literature sourced from PubMed and Cochrane libraries

The first aspect of the study was to review recent literature to address the ongoing debate with regard to the preference for TAPP versus TEP hernia repair techniques. This would help to address key principles that have been identified, matters that have been concluded, and issues still being debated amongst the community. A review of the literature was approached systematically as evidenced by the PRISMA algorithm shown in Figure 1.

The second limb of the study involved a comparative analysis of all inguinal hernia surgeries completed within a total of three separate healthcare settings, namely Pilgrim and Lincoln Hospital, Lincolnshire, United Kingdom and Mediclinic Parkview Hospital, Dubai, UAE. Surgeries were reviewed and followed during a two-year period starting from January 2020 to December 2022.

Based on inguinal hernia prevalence, the incidence of surgery being performed in both countries and the total population size of the cities represented by each healthcare center a total of 95 procedures for each technique (TAPP and TEP) was determined as the minimum number of patients required to ensure statistically significant conclusions could be drawn when analyzing the data.

Surgical cases were selected based on strict inclusion criteria. Cases deemed appropriate for inclusion were age 18 to 65 years, both male and female sex, presentation for primary unilateral inguinal hernia as an elective procedure which is due to be performed by a general surgeon of greater than five-year experience with either TAPP or TEP inguinal hernia repair.

Cases were subsequently excluded if any of the following criteria were found age greater than 65 years, evidence of bilateral inguinal hernia or femoral hernia, previous history of recurrence and/or previous inguinal hernia surgery, ASA grade 4 or greater and if there was evidence of a clinically incarcerated or strangulated inguinal hernia. Each surgeon included in the trial was assigned to a single laparoscopic technique. This was determined based on which technique was performed more frequently and all additional procedures performed using alternative techniques were excluded.

Data collection regarding postoperative analgesia, operative complications, and postoperative follow-up records were obtained via the electronic health record (EHR) at each facility. The data were compared using the Chi-squared and unpaired Student’s t-test for qualitative and quantitative parameters.

## Results

From the comparative analysis of inguinal hernia repair across three surgical units, a total of 1,321 hernia repairs were conducted of which a total of 279 cases fell within the inclusion criteria. Of these cases, 38% were performed using the TEP method (n=106), while 62% were performed using the TAPP method (n=173). As stated previously each of the 279 cases were uncomplicated, primary, unilateral inguinal hernias of similar patient demographics.

Demographic distribution

The mean age for the population was 45 ± 11.2 years (range = 21-65 years). The mean age of patients within the TEP subgroup was 42.1 ± 5.6 years (range = 21 - 59 years) and the TAPP subgroup was 46.8 ± 16.0 years (range = 22 - 65 years). There was a total of 24 (8.6%) female patients of which 17 were performed as TAPP procedures. There was no statistically significant difference in terms of distribution between the subgroups with regards to age and sex which may be seen in Table 1.

**Table 1:** Patient demographic of each hernia repair subgroup categorised by gender and age group

TAPP Subgroup			TEP Subgroup		
Age	Male	Female	Age	Male	Female
18-19	0	0	18-19	0	0
21-30	5	0	21-30	1	0
31-40	17	0	31-40	12	2
41-50	72	1	41-50	42	3
51-60	30	13	51-60	35	2
61-70	32	3	61-65	9	0
Total	156	17	Total	99	7

Postoperative pain

Patients were requested to complete a visual analogue pain score (VAS) at one and 24 hours postoperatively as well as at a one-week surgical follow up to determine the level of postoperative pain. There was a statistically significant difference in pain scores between the subgroups at one and 24 hours without any significant difference at the one-week follow-up. The data are outlined in Table 2.

**Table 2:** Postoperative pain scores assessed using VAS system comparing TAPP vs TEP hernia repair techniques at predetermined intervals.

	TAPP Repair	TEP Repair	P-value
Pain Score 1 hour (mean ± SD)	2.88 ± 0.34	1.67 ± 0.45	< 0.05
Pain Score 24 hours (mean ± SD)	3.14 ± 0.73	1.97 ± 0.31	< 0.05
Pain Score 1 week (mean ± SD)	1.32 ± 0.23	1.07 ± 0.19	0.387

Of note, within both subgroups pain was noted to be higher 24 hours post operatively in comparison to the one-hour postoperatively results, however across both enquiries the patient undergoing TEP hernia repair reported significantly less pain with controlled post operative anesthetic analgesia (oral opioids)

Length of hospital stay

Of all 279 patients, only 13 required inpatient care beyond 24 hours. As a result, no statistically significant data or comparative analysis could be performed between the two subgroups and at this point there shows no difference in technique with regards to length of hospital stay. Of the 13 patients requiring extended length of stay, the maximal duration was 72 hours due to concerns regarding post operative pain and scrotal edema in a single patient. Post operative complications outside of hernia recurrence were not assessed in this analysis but none of the previously mentioned 13 patients suffered significant morbidity or required readmission prior to their one-week surgical follow-up.

Recurrence rate

**Figure 2:**
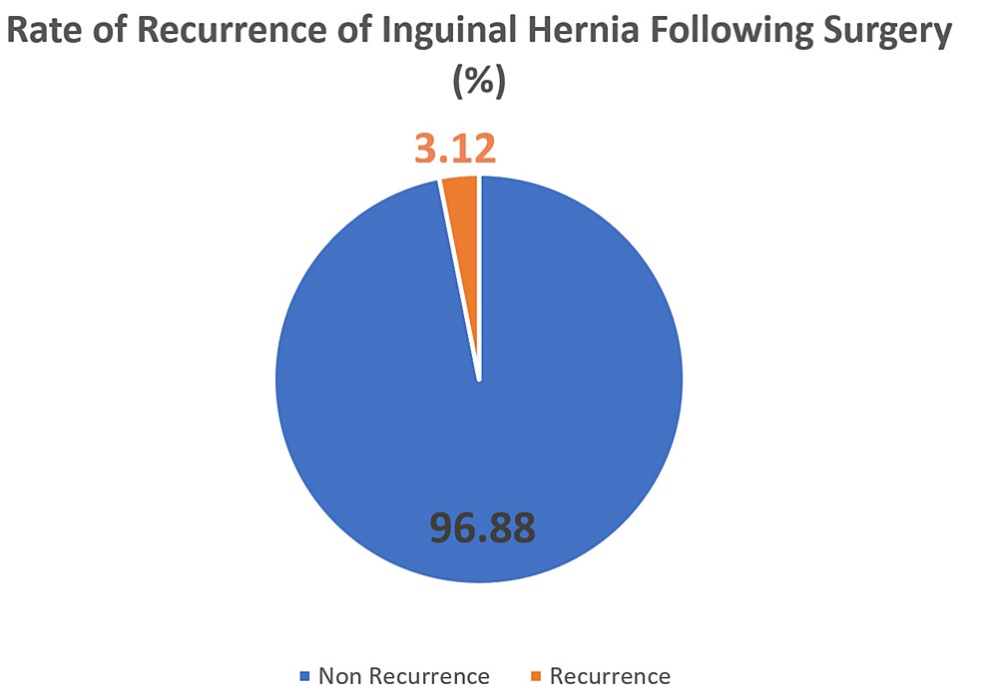
Pie chart showing the total recurrence rate across both subgroups of primary inguinal hernia repair at one year

**Figure 3:**
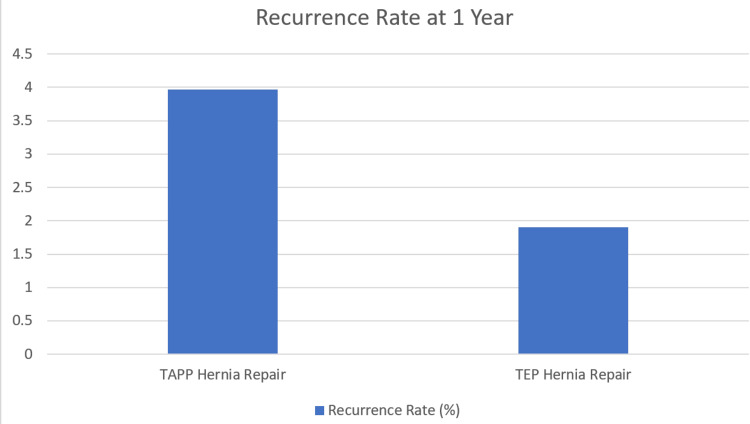
Bar chart showing the recurrence rate of primary inguinal hernia at one year following each repair subgroup

Of the 279 patients initially analyzed a total of 23 were lost to follow up beyond their one-week surgical planned outpatient consultation. These 23 patients were either unable to be contacted due to missing clerical information or had passed away due to causes unrelated to surgical interventions. This left a total of 256 patients (TEP, n=105 and TAPP, n= 151) all of whom were contacted between six months to one year following their surgical date. Of these 256 patients, total of eight patients (3.1%) had experienced recurrence of inguinal hernias within one year following surgery which can be seen in Figure 2. Of these eight patients, six (3.97%) had been a part of the TAPP repair group and two (1.9%) had been a part of the TEP repair group as shown in Figure 3. There was no statistically significant difference in recurrent rates between repair groups (p =0.213).

Patient satisfaction

**Figure 4:**
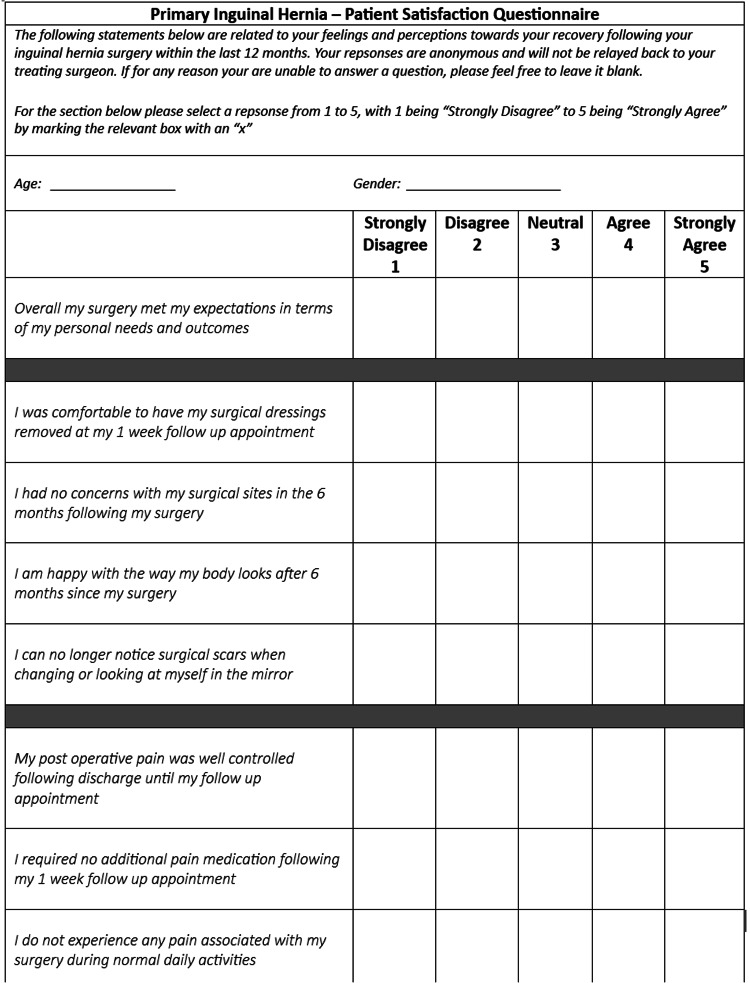
Extract of the patient satisfaction questionnaire used to assess patient responses relevant to cosmetic outcomes, pain scores and overall satisfaction

An attempt was made to quantify patient satisfaction. By using a simple questionnaire in which patient responses were categorized numerically from 1-5, with 1 being an extreme negative connotation and 5 being an extreme positive connotation, we were able to gather a data set of numerical values centered around a 10-part questionnaire. The questionnaire focused on aspects such as overall satisfaction with surgical outcome, post operative pain control, chronic pain concerns and cosmetic outcome at six months. An extract of the questionnaire may be seen in Figure 4. There was limited variation in the collected data and for the purpose of representing the majority, the patients who experienced hernia recurrence were removed from the sample.

Overall satisfaction was classified as a score of 4-5, whereas dissatisfaction as classed as a score of < 4. Of the TAPP subgroup a total of 138 patients (97.1%) expressed satisfaction compared to 101 patients within the TEP subgroup (98.1%). Cosmetic outcome was the only metric which showed an appreciable difference in outcome across the female demographic of patients. Of the seven female patients who underwent a TEP procedure all reported a cosmetic outcome of four or five (100%) whereas the TAPP subgroup reported a cosmetic satisfaction of only 11 out of 17 (64.7%).

Of the five variables set out to be investigated; length of hospital stay, post operative pain, patient satisfaction, recurrence rate and cosmetic outcome, there was shown to be a statistically significant difference in the following.

· Reduced postoperative pain at 1 hour in the TEP subgroup compared to TAPP subgroup (1.67 ± 0.45, vs 2.88 ± 0.34, p: < 0.05).

· Reduced postoperative pain at 24 hours in the TEP subgroup compared to TAPP subgroup (1.97 ± 0.31, vs 3.14 ± 0.73, p: < 0.05).

Additionally noticeable difference in cosmetic outcome amongst female patients was reported; however, due to the small sample size of 24 patients within the study no conclusions may be drawn. Improved cosmetic outcome amongst female patients undergoing TEP inguinal hernia repair with satisfaction of 100% compared to 64.7% of female patients undergoing TAPP inguinal hernia repair.

## Discussion

As stated previously, the intent of the team was to discern if it would be warranted to advocate for a policy in which surgeons were encouraged to adopt a specific inguinal hernia repair technique based on the evidence. From the evidence provided and that found in the literature, it does not appear feasible to be able to suggest such a policy. From the data collected the TEP inguinal hernia result appears to be superior in outcome however not to a degree that it is definitive or significant. This benefit needs to be weighed against the learning curve of this procedure as shown in the literature [5]. The positive surgical outcomes reflected in this study have been achieved by surgeons with great experience with this technique and similar outcomes are likely not to be achieved with surgeons familiar with TAPP inguinal hernia repair being forced to adopt a new technique.

From a review of the literature, it was clear that there was no definitive treatment outlined as the clear superior method when comparing TAPP with TEP hernia repair [6,7]. Advocates for TEP would site the amenability for bilateral hernia repair and the reduction of representation and improved cosmetic outcome [8], whereas advocates for TAPP would site the reduced operative time [9] as well as the steep learning curve for TEP and the related intra and post-operative complication rates with TEP procedure in the hands of newly practicing surgeons [10].

A final point of debate that was circulated amongst the team at the time of the study was the evolution of surgical techniques. At the commencement of laparoscopic surgery more than 50 years prior studies concluded poorer or equal outcomes in newly discovered laparoscopic techniques when compared to their open surgical counterparts [11,12]. These studies cited surgeon inexperience, technician and equipment error, and cost as reasons to abandon laparoscopic techniques which we now know to be far superior in recent literature [13].

Should the same process of thought not be applied today? Should the new techniques be advocated to improve the outcome of surgical patients if currently there is no literature to suggest harm or risk to our patients? If this is true, then it is logical to suggest that TEP inguinal hernia repair is the technique of choice. Once the learning curve is overcome the overall surgical outcomes have shown to be statistically significant over TAPP inguinal hernia repair [14]. However, what is the acceptable risk to our patients and is there an acceptable level of morbidity that we can tolerate while overcoming this obstacle of complexity? During the course of this study, literature has already emerged comparing outcomes of TAPP/TEP when compared to robotic minimally invasive procedures [15]. Will advocating for TEP inguinal hernia repair improve the outcome of patients or could anyone apply the same logic to assert that time and effort should now be placed on robotic surgery for the betterment of surgical patient outcomes?

The study was unfortunately limited by its size. Given the similarities in outcome discovered during the course of the comparison a far larger sample size would be required in order to determine if the differences noted within the study had any statistical significance. The shorter duration of the study also did not allow for long-term follow which would have given the authors the opportunity to assess each subgroup for the possibilities of chronic pain and worsening recurrence rates at three- and five-year intervals.

## Conclusions

The comparative analysis would suggest that surgeons capable of performing a TEP inguinal hernia repair should continue to do so; however, the marginal nature of the results would also encourage those performing TAPP procedures to continue as there is no significant data to show any disadvantage to the patient in the long term. Overall, the study is unable to suggest alterations to ongoing practices, however, based on the evidence collected it would be suggested that any surgeon with the means to perform either TEP or TAPP inguinal hernia procedures should favor a TEP repair if not otherwise contra-indicated.

The findings of this study now give the community the ability to assess the merits of each inguinal hernia repair technique. If it is confirmed that each technique is valid and with minimal risk to the patient then it may be investigated which technique may benefit specific patient demographics. Further work can be conducted to determine if favorable outcomes are obtained for each technique in the obese, geriatric, or poly-morbid populations for example.
